# Enzymatic Hydrolysis Optimization for Preparation of Sea Cucumber (*Holothuria scabra*) Hydrolysate with an Antiproliferative Effect on the HepG2 Liver Cancer Cell Line and Antioxidant Properties

**DOI:** 10.3390/ijms24119491

**Published:** 2023-05-30

**Authors:** Supansa Saiwong, Narongchai Autsavapromporn, Thanyaporn Siriwoharn, Charin Techapun, Sutee Wangtueai

**Affiliations:** 1 Faculty of Agro-Industry, Chiang Mai University, Chiang Mai 50100, Thailand; supansa_saiw@cmu.ac.th (S.S.); thanyaporn.s@cmu.ac.th (T.S.); charin.t@cmu.ac.th (C.T.); 2 Division of Radiation Oncology, Department of Radiology, Faculty of Medicine, Chiang Mai University, Chiang Mai 50200, Thailand; narongchai.a@cmu.ac.th; 3 College of Maritime Studies and Management, Chiang Mai University, Samut Sakhon 74000, Thailand

**Keywords:** sea cucumber, protein hydrolysate, antioxidant activity, antiproliferative activity

## Abstract

The sea cucumber body wall was subjected to enzymatic hydrolysis using papain. The relationship between the enzyme concentration (1–5% *w*/*w* protein weight) and hydrolysis time (60–360 min) and the degree of hydrolysis (DH), yield, antioxidant activities, and antiproliferative activity in a HepG2 liver cancer cell line was determined. The surface response methodology showed that the optimum conditions for the enzymatic hydrolysis of sea cucumber were a hydrolysis time of 360 min and 4.3% papain. Under these conditions, a 12.1% yield, 74.52% DH, 89.74% DPPH scavenging activity, 74.92% ABTS scavenging activity, 39.42% H_2_O_2_ scavenging activity, 88.71% hydroxyl radical scavenging activity, and 9.89% HepG2 liver cancer cell viability were obtained. The hydrolysate was produced under optimum conditions and characterized in terms of its antiproliferative effect on the HepG2 liver cancer cell line.

## 1. Introduction

Oxidation processes in food or material degradations are mainly caused by a reaction of free radicals. Free radicals also cause cellular and tissue damage, leading to human disorders such as aging-associated diseases, cancer and inflammatory diseases, and cardiovascular diseases [1]. Active oxygens and free radicals are produced during human respiration and in other aerobic organisms. They are unstable compounds that rapidly react with other substances and molecules in the body [2]. Reactive oxygen species (ROS) represent an important type of free radicals in various biological systems. ROS usually contain hydrogen peroxide (H_2_O_2_), hydroxyl radicals (OH·), superoxide radicals (O_2_), and singlet oxygen (O_2_) [3]. Although the human body has a mechanism for preventing these free radicals from damaging the cells and tissues, different types of human diseases may still occur when there is an imbalance between the free radicals and endogenous antioxidants due to oxidative stress in the body [4]. Cancer is one of the leading causes of human mortality, and oxidative stress is involved in the pathogenesis of inflammatory cancers [5]. At present, cancer treatment uses one or more methods, such as surgery, chemotherapy, radiotherapy, and medicine. However, many studies have indicated that these methods could damage normal cells or organs [6]. Therefore, antioxidants have been proposed as suitable candidates for preventing and providing therapy for a variety of health disorders caused by ROS-related diseases, especially cancer [3]. Oxidative chain reactions of ROS are terminated by the free radical scavenging function of antioxidants. Synthetic antioxidants have strong antioxidant activities but low toxicity and side effects [7]. Hence, researchers’ attention is being drawn toward natural marine sources with antioxidant and anticancer activities, such as protein hydrolysates from Indian mackerel waste [3], blood clam muscle [5], and rainbow trout skin [2].

Marine protein hydrolysates and peptides produced by enzyme hydrolysis demonstrate significant antioxidative activities against free radicals and could potentially be used as alternatives to synthetic antioxidants in food and the human body [5]. Sea cucumber contains high amounts of protein and low levels of lipids and is a highly valuable marine animal [8]. Sea cucumber is consumed in Asian countries as a culinary delicacy and supplementary food [9,10] and has been researched due to its bioactive compounds, such as polysaccharides, triterpene glycosides, saponin, phenols, and peptides, as well as its various antimicrobial, anti-inflammatory, antithrombotic, antidiabetic, anti-obesity, antioxidant, and anticancer properties [11].

Therefore, the objective of this research was to investigate the optimum conditions for the preparation of the sea cucumber hydrolysate with antioxidant and anticancer activities using papain hydrolysis. The response surface methodology with a face-centered central composite design was used to determine the effect of the enzyme concentration and hydrolysis time on the yield, degree of hydrolysis (DH), antioxidant activities, and antiproliferative activity of a HepG2 liver cancer cell line. This is a preliminary study for optimizing the preparation of the sea cucumber hydrolysate with antioxidant properties that also has an antiproliferative effect on a HepG2 liver cancer cell line.

## 2. Results and Discussion

### 2.1. Chemical Composition of Sea Cucumber

The chemical compositions of the plain dried and slurry samples of the gutted and skinned sea cucumber (*Holothuria scabra*) were determined and are shown in Table 1. Crude protein and moisture are the main components of dried sea cucumber and sea cucumber slurry, respectively. Li et al. [10] reported that the main component of protein in the sea cucumber body wall is collagen, which can be converted into a functional bioactive compound. Sea cucumber contains low levels of lipids and high amounts of ash. Previous studies show that dried sea cucumber contains 15% moisture, 65% crude protein, 0.6% lipid, and 12% ash content [8], while the study by Li et al. [10] shows that the ranges of protein, lipid, and ash contents are 52.4–54.2%, 0.21–0.41%, and 14.0–30.7%, respectively. The content of crude protein in sea cucumber slurry (Table 1) was used to calculate the enzyme concentration for the addition of enzymes in the hydrolysis process.

### 2.2. Optimization of Sea Cucumber Hydrolysis Conditions

The optimization of sea cucumber hydrolysis conditions was conducted using response surface methodology with a face-centered central composite design. The results of all 11 experimental units and 3 replicates are shown in Table 2. The yield (Y_1_) ranged from 6.69% to 12.75%, DH (Y_2_) ranged from 79.58% to 85.22%, DPPH radical scavenging activity (DPPH, Y_3_) ranged from 79.75% to 91.66%, ABTS radical scavenging activity (ABTS, Y_4_) ranged from 59.88% to 69.33%, H_2_O_2_ radical scavenging activity (H_2_O_2_, Y_5_) ranged from 18.66% to 41.7%, hydroxyl radical scavenging activity (OH, Y_6_) ranged from 73.03% to 84.56%, and antiproliferative activity in the HepG2 liver cancer cell line, referred to as %HepG2 liver cancer cell viability (cell viability, Y_7_), ranged from 2.61% to 5.14%. The real variables of the responses were used to generate full quadratic mathematical models. The influence of two hydrolysis factors, X_1_ (hydrolysis time: min) and X_2_ (papain concentration: %*w*/*w* protein), on all responses for the preparation of the sea cucumber hydrolysate were determined and are shown in Table 2.

#### Response Surface Model Generation

Response surface models were created to observe the effect of hydrolysis conditions (X_1_ and X_2_) on responses (Y_1_ to Y_7_), for which a multiple-regression analysis was conducted with a full-quadratic response surface, as shown in Table 3. The constants, coefficients, and *p*-values of the independent variables and interactions for all responses are shown in Table 4. Most of the models were highly significant (*p* ≤ 0.01), except for the models of OH· (Y_6_) and cell viability (Y_7_), which were significant with a confidence interval of 95% (*p* ≤ 0.05). However, the lack of fit for all models was not significant (*p* > 0.05) and had high coefficients of determination (*R*^2^) within a range of 0.84 to 0.97. These models were appropriate for addressing a correlation among the factors of interest with a high certainty. A significant model with a high *F*-value for the lack of fit and a high *R*^2^ is able to accurately determine the influence of the independent variables on the observed data [12].

### 2.3. Response Surface Plots

**Figure 1:**
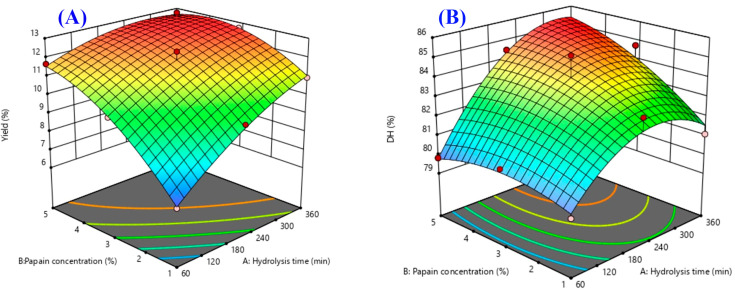
The 3D and contour plots of the hydrolysis time (X_1_: min) against the concentration of papain (X_2_: %) for the yield ((**A**): %) and DH ((**B**): %).

**Figure 2:**
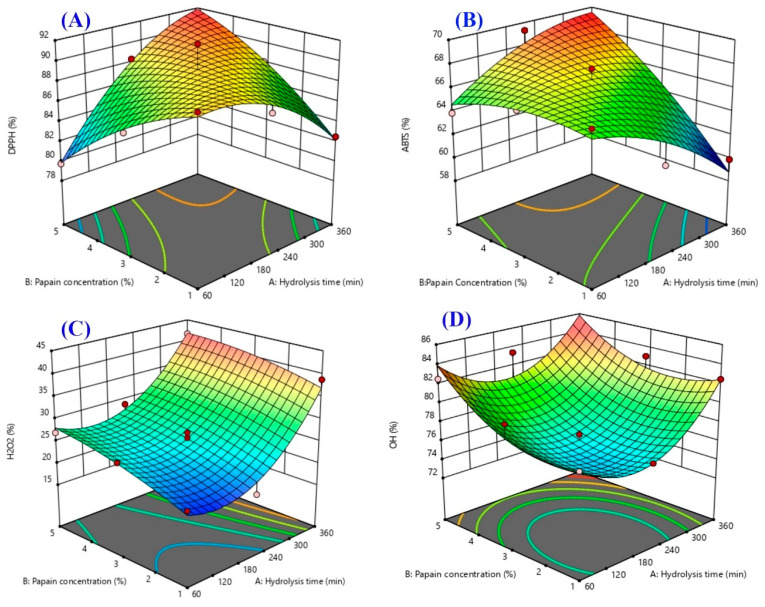
The 3D and contour plots of the hydrolysis time (X_1_: min) against the concentration of papain (X_2_: %) for the antioxidant activities, such as DPPH radical scavenging activity (**A**), ABTS radical scavenging activity (**B**), H_2_O_2_ scavenging activity (**C**), and OH· radical scavenging activity (**D**).

**Figure 3:**
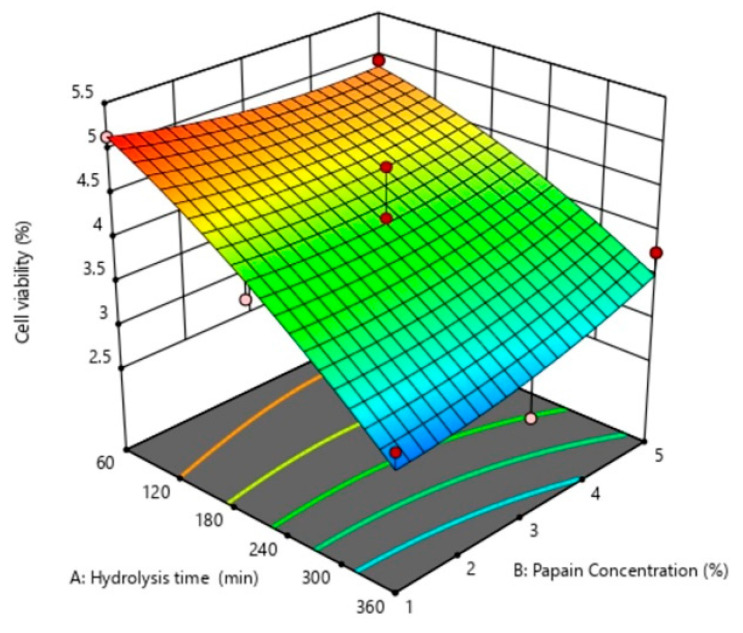
The 3D and contour plots of the hydrolysis time (X_1_: min) against the concentration of papain (X_2_: %) for antiproliferative activity in the HepG2 liver cancer cell line, reported as HepG2 liver cancer cell viability (%).

Response surface plots were drawn as three-dimensional and contour plots (Figure 1, Figure 2 and Figure 3), which show the relationship between two factors (hydrolysis time: X_1_ and papain concentration: X_2_) and the responses, including yield (%), DH (%), DPPH radical scavenging activity (%), ABTS radical scavenging activity (%), H_2_O_2_ radical scavenging activity (%), OH· radical scavenging activity (%), and antiproliferative activity in the HepG2 liver cancer cell line (%HepG2 cell viability). 

**Figure 4:**
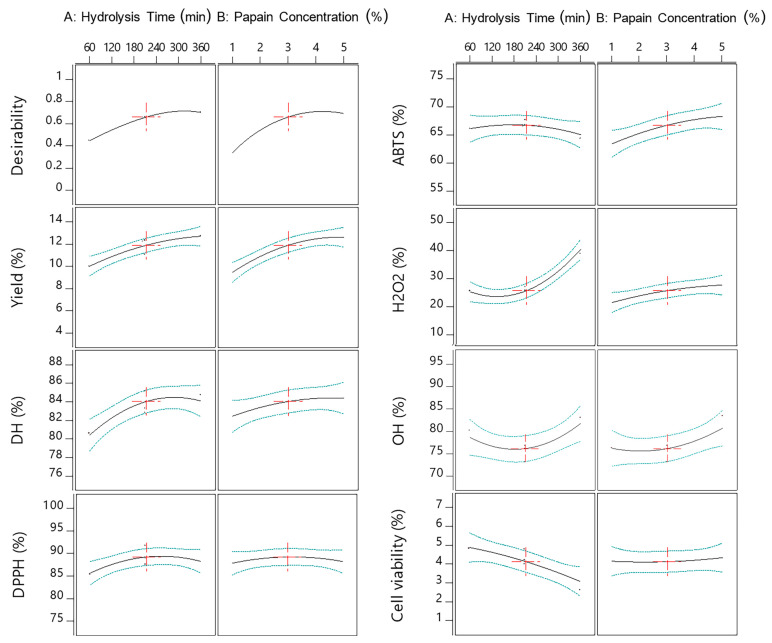
Plots for the significant main effects of the hydrolysis time (X_1_) and papain concentration (X_2_) on DH, yield, anticancer activity (%HepG2 cell viability), DPPH, ABTS, H_2_O_2_, and OH· radical scavenging activity. The red crosses show the center point of the factors.

**Figure 5:**
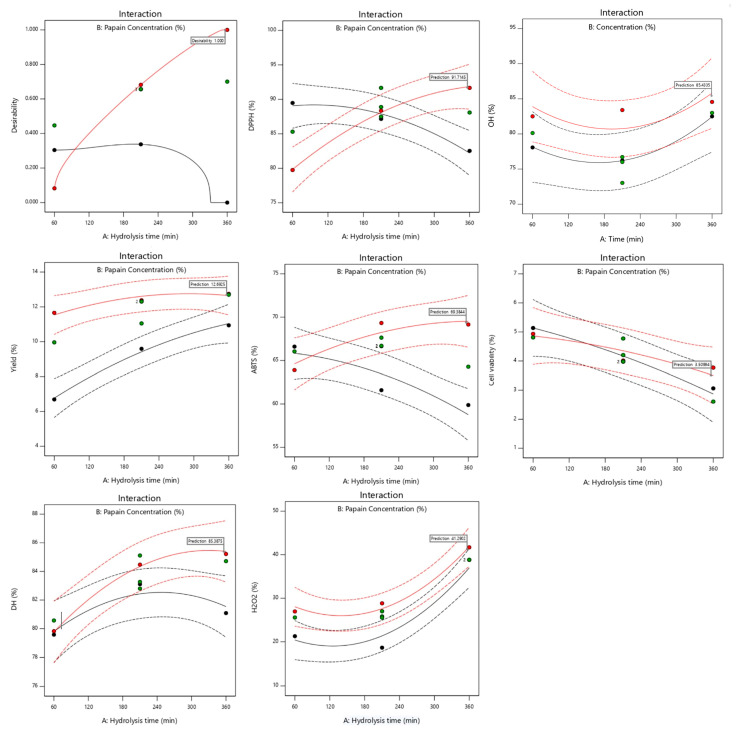
Plots for the interaction effects of the hydrolysis time (X_1_) and papain concentration (X_2_) on DH, yield, anticancer activity (%HepG2 cell viability), DPPH, ABTS, H_2_O_2_, and OH· radical scavenging activity.

Figure 1A shows the effects of the two factors (X_1_ and X_2_) on the yield. Both factors had a significant main effect (Figure 4) and interaction effect (Figure 5) on the hydrolysate yield. The increase in X_1_ and X_2_ also caused an increase in the hydrolysate yield of sea cucumber. This may be due to the higher degree of peptide breakdown caused by the addition of water molecules to the protein structure of the substrate, converting it to short or long peptides, and thus obtaining a higher hydrolysate yield [13]. According to Doungapai et al. [8], Mongkonkamthorn et al. [14], and Halim and Sarbon [15], an increase in the enzyme concentration and hydrolysis time affects the enzyme’s reaction with the protein substrate, resulting in more hydrolysate products. Moreover, the yield of the protein hydrolysate is influenced by the protein substrate, enzymes, and hydrolysis conditions, such as pH and temperature [16].

Regarding the DH, only X_1_ had a main effect (Figure 4), while no interaction effect was observed between X_1_ and X_2_ on the DH (Figure 5). The indicator of enzymatic protein hydrolysis could be evaluated via the DH value, which shows the ability of the enzyme to breakdown peptide bonds in the protein structure [17]. The DH increased with the increasing hydrolysis time, while the highest DH was observed when the hydrolysis time was approximately 240 min (Figure 1B). This result was similar to previous studies by Doungapai et al. [8] and Upata et al. [13]. These authors reported that the hydrolysis time had a significant main effect on the DH regarding papain hydrolysis in sea cucumber or jellyfish. In another study, Hsu et al. [18] found that the hydrolysis of tuna dark muscle using papain (specific activity 3.8 U/mg) achieved the highest DH at a 360 min hydrolysis time. However, the DH value rapidly increased at the beginning of the enzyme reaction, and the rate of the increasing DH decelerated at higher DH values and tended to stabilize [19]. This might be due to the higher availability of enzyme active sites at the start of the enzyme hydrolysis reaction that could initially react and cause a higher number of peptide bonds to breakdown [20]. 

The effects of the independent variables (X_1_ and X_2_) on the antioxidant properties (DPPH, ABTS, OH·, and H_2_O_2_ scavenging activities) are shown in Figure 2A–D. The effects of the two factors on the DPPH scavenging activity of the sea cucumber hydrolysate are shown in Figure 2A. Although no significant main effects of X_1_ and X_2_ on DPPH radical scavenging activity were found (Figure 4), an interaction effect was observed (Figure 5). The DPPH radical scavenging activity of the protein hydrolysate demonstrated an ability to convert DPPH radicals into a stable product and terminate the chain reactions of the radicals [21]. DPPH is a widely used radical applied to evaluate the free radical scavenging activity of antioxidative compounds as a free radical scavenger or hydrogen donor [22]. Both the enzyme concentration and the hydrolysis time could affect the DH associated with the antioxidant properties of the hydrolysate, as previously reported in the protein hydrolysates of tuna dark meat [14], Indian mackerel waste [3], and jellyfish [13]. An increase in the hydrolysis time and enzyme concentration resulted in a higher DH and a lower molecular weight of peptides obtained with higher antioxidative activity [23]. With a greater DH, a breakdown of the interior peptide bonds in the original protein structures generates small peptides (short- or medium-chain), causing the release of higher hydrophobic amino acids in peptide chains that may react to inhibit the DPPH free radicals [15,24]. The results for the ABTS radical scavenging activity of the sea cucumber hydrolysate are shown in Figure 2B. X_2_ had a significant main effect on ABTS radical scavenging activity (Figure 4), while interaction effects between X_1_ and X_2_ were observed, as shown in Figure 5. The ABTS radical scavenging activity of the protein hydrolysate indicated the ability of the hydrolysate to be an effective electron or hydrogen donor to react with unstable ABTS free radicals, converting them to a stable product and leading to a termination of the free radical reaction [5]. In this study, the enzyme concentration and the interaction between the hydrolysis time and the enzyme concentration significantly influenced the ABTS radical scavenging activity of the sea cucumber hydrolysate. The increase in the enzyme concentration caused an increase in ABTS radical scavenging activity. This was similar to the previous study of the white shrimp protein hydrolysate, which found that hydrolysates with a higher DH have a higher ability of capturing ABTS free radicals [25]. Additional studies also reported that the hydrolysis time and enzyme concentration had a significant effect on the antioxidant activities of hydrolysates in duck egg, tuna blood, and jellyfish [13,17,26].

The effects of X_1_ and X_2_ on the H_2_O_2_ scavenging activity of the sea cucumber hydrolysate are shown in Figure 2C. Both independent variables had a significant main effect on H_2_O_2_ scavenging activity (Figure 4), but there was no interaction effect (Figure 5). The sea cucumber hydrolysate had a higher H_2_O_2_ scavenging activity with the increasing hydrolysis time and enzyme concentration. Je et al. [27] reported that the electron donation ability of antioxidant compounds contributes to their H_2_O_2_ scavenging activity. In general, H_2_O_2_ is a weak oxidizing agent that cannot directly react in the initiated oxidation process of lipids due to its lower reduction potential than that of unsaturated fatty acids. However, H_2_O_2_ indirectly contributes to the oxidation process of lipids as a precursor to the generation of hydroxyl radicals, a strong initiator in the lipid oxidation process [28]. Je et al. [27] explained that H_2_O_2_ is a reactive non-radical that can penetrate through biological membranes and damage cells. 

The effects of X_1_ and X_2_ on the hydroxyl (OH·) radical scavenging activity of the sea cucumber hydrolysate are shown in Figure 2D. Both factors had significant main effects, without the interaction effects for OH· scavenging activity (Figure 4 and Figure 5). The OH· scavenging activity increased with the increasing enzyme concentration and hydrolysis time (Figure 4). The OH· radical is a major ROS with the strongest chemical reactivity. It easily reacts with the membrane components, cellular proteins, and DNA of the cells, thereby causing cell toxicity [27]. This finding is consistent with Zhuang et al. [29], who found that the OH· scavenging activity of jellyfish hydrolysates increased as the enzyme concentration increased. Je et al. [27] also reported that a longer hydrolysis time obtained smaller peptides, which had a significant effect on the OH· radical scavenging of the tuna liver hydrolysate. 

The effects of X_1_ and X_2_ on the cell viability of the HepG2 liver cancer cell line of the sea cucumber hydrolysate are shown in Figure 3. Only the X_1_ variable had significant main effects on cell viability (Figure 4), while there was no interaction effect (Figure 5). The hydrolysis time had an increasingly significant effect as cell viability decreased. This study is consistent with the results of Hsu et al. [18]. These authors reported that the hydrolysis time influenced the antiproliferative activity in cancer cells of the tuna dark meat hydrolysate using papain, in which the strongest antiproliferative activity was obtained at hydrolysis times of 60 and 120 min. Chi et al. [5] reported that the hydrolysate of bloody clam meat exhibited good free radical scavenging activities and promoted anticancer activity by inducing cell apoptosis and inhibiting cell proliferation. The anticancer activities of hydrolysates or peptides are associated with their antioxidant activities. Ishak and Sarbon [30] reported that small peptides derived from fish-processing byproducts have good anticancer activity, especially MW, ranging from 300 to 1950 Da. This is because the lower MW peptides have greater molecular mobility and diffusivity than those of higher MW peptides. 

### 2.4. Optimization of Multiple Responses and Model Validation

The optimal conditions for the preparation of the sea cucumber hydrolysate with antioxidant and anticancer activities were determined using a function of the Design Expert statistical program. The dependent variable parameters (yield, DH, DPPH, ABTS, H_2_O_2_, and OH·) were set as maximum goals, while the cell viability of HepG2 was set as a minimum goal. The composite desirability and the various values of predicted and experimental responses are shown in Table 5, assuming that most of the responses were in agreement with the predicted values. The optimal conditions to obtain a maximum yield, as well as optimal antioxidant and antiproliferative effects on the HepG2 liver cancer cell line properties, were determined as a 4.3% papain concentration and a 360 min hydrolysis time. The sea cucumber hydrolysate was prepared under optimum conditions to validate the production process via a study of its concentration effect on the antiproliferative activity in the HepG2 liver cancer cell line.

### 2.5. The Effect of the Sea Cucumber Hydrolysate Concentration on Anticancer Activity

**Figure 6:**
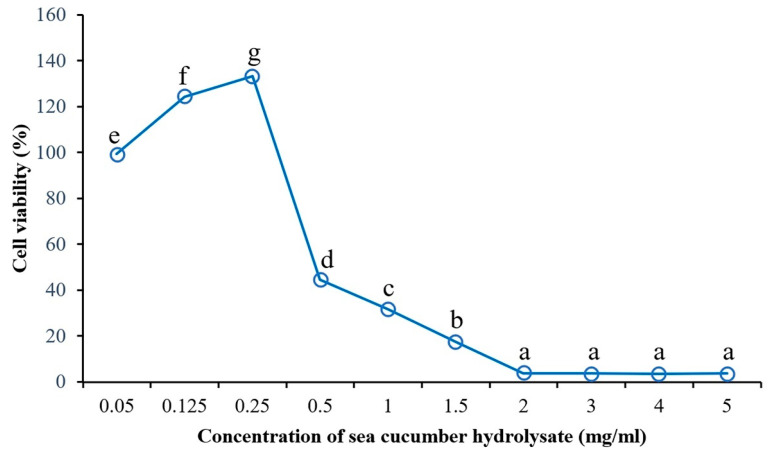
Cell viability of the HepG2 liver cancer cell line treated with the sea cucumber protein hydrolysate for 24 h at various concentrations. Different letters in the same column indicate statistical differences (*p* ≤ 0.05).

The sea cucumber hydrolysate was tested for cytotoxicity effects on HepG2 cells, referred to as anticancer activity, using the MTT assay. The effect of the hydrolysate concentration on HepG2 cell viability was studied at various concentrations (0.05–5.0 mg/mL). An increase in HepG2 cell viability was observed at concentrations ranging from 0.05 to 0.25 mg/mL, and cell viability subsequently decreased. On the other hand, cell survival decreased as the concentration of the sea cucumber hydrolysate increased (Figure 6). However, HepG2 cell viability was not significantly different (*p* > 0.05) when treated with concentrations ranging from 2.0 to 5.0 mg/mL and a range of cell viability from 3.52% to 3.99%. According to a previous study of an eel protein hydrolysate on a human breast cancer MCF-7 cell line, cell survival significantly decreased with increased eel protein hydrolysate concentrations of 5 kDa and 3 kDa [31]. Song et al. [32] also reported that gecko crude peptides significantly inhibited HepG2 cell proliferation, depending on the concentration and the treatment time. In addition, the blood clam protein hydrolysate indicated cytotoxicity in cancer cells in a dose-dependent manner, with IC_50_ in the range of 1.99 to 2.53 mg/mL. The hydrolysate contained higher hydrophobic amino acids in peptide chains, leading to an increased interaction between the blood clam protein hydrolysate and cancer cells [5].

## 3. Materials and Methods

### 3.1. Raw Materials and Preparations

Plain dried, gutted, and skinned sea cucumber (*Holothuria scabra*) samples were obtained from a local fishery at Pu Island (Krabi Province, Thailand). They were packed in a plastic bag and kept in an insulated box during transportation to the laboratory of the College of Maritime Studies and Management, Chiang Mai University, Samut Sakhon Province. Upon arrival, the samples were packed in zip-lock polyethylene bags and stored at −18 to −20 °C until use (<3 months). Before use, the samples were rehydrated in distilled water. In brief, dried sea cucumbers were cut into small pieces (5–6 mm squares) by hand using a knife, soaked in distilled water at a ratio of 1:2 (w:v) for 1 h at room temperature (25 ± 1°C), left to drain in a plastic basket for 5 min, homogenized (Cole-Parmer T-25 digital ULTRA-TURRAX, Wertheim, Germany) with distilled water (1:5 *w*/*w*) to obtain a slurry of sea cucumber, packed in zip-lock polyethylene bags (300 g/bag), and kept at −18 to −20 °C until use (<3 months).

### 3.2. Chemicals and Enzymes

Papain (specific activity of ≥30,000 U/mg), 2,2-diphenyl-1-picrylhydrazyl (DPPH), 2,2′-azino-bis(3-ethylbenzothiazoline-6-sulfonic acid (ABTS), and 2,4,6-trinitrobenzenesulfonic acid solution (TNBS) were purchased from Sigma-Aldrich (St. Louis, MO, USA). Hydrogen peroxide was purchased from Merck (Darmstadt, Germany). Disodium hydrogen phosphate dihydrate was purchased from RCI Labscan (Bangkok, Thailand). Sodium dihydrogen phosphate dihydrate, 1,10-phenanthroline, and ferrous sulfate heptahydrate were purchased from QReC (Quality Reagent Chemical, Auckland, New Zealand). Peroxidase (horseradish, specific activity of 24 U/mg) was purchased from Thermo Fisher Scientific (Waltham, MA, USA). All other reagents used in the experiment were of analytical grade.

### 3.3. Enzymatic Hydrolysis of Sea Cucumber

Next, 300 g of sea cucumber slurry (Section 3.1) was thawed under running tap water, placed in a 500 mL Erlenmeyer flask, and then heated at 90 °C for 15 min to inactivate the endogenous enzymes. For the hydrolysis process, papain was added to the sea cucumber slurry at concentrations of 1–3% (*w*/*w* protein) and hydrolyzed in a shaking water bath (Memmert WNB45, Schwabach, Germany) at 50 °C for 60–360 min. After hydrolysis, the hydrolysate solution was heated at 90 °C for 15 min to inactivate the enzymes, cooled under running tap water, and centrifuged at 2100× *g* for 10 min. The supernatant was then collected and freeze-dried (GFD-3H, Grisrianthong, Samut Sakorn, Thailand) to obtain a sea cucumber hydrolysate powder.

### 3.4. Analyses

#### 3.4.1. Chemical Composition of Sea Cucumber

Moisture, protein, fat, and ash contents of sea cucumber were determined according to the method of AOAC [33], and were 934.01, 954.01, 991.36, and 942.05 method number, respectively. The nitrogen content was converted to crude protein with a conversion factor of 6.25.

#### 3.4.2. Yield

The dried yield of the sea cucumber hydrolysate was calculated according to the method of Mongkonkamthorn et al. [17] using the following equation:

(1) $$Yield(\%)=\frac{weight\;of\;dried\;sea\;cucumber\;hydrolysate\;(g)}{weight\;of\;rehydrated\;sea\;cucumber\;(g)}\times 100$$

#### 3.4.3. Degree of Hydrolysis (DH)

The DH of the sea cucumber hydrolysate was determined according to the method of Mongkonkamthorn et al. [17]. A total of 125 µL of the samples was mixed with 2 mL of 0.2 M sodium phosphate buffer (pH 8.2), prior to the addition of 1 mL of 0.01% TNBS. The mixtures were incubated in a water bath at 50 °C for 30 min in the dark. The reaction of the mixtures was terminated via the addition of 2 mL of 0.1 mmol/L sodium sulfate, and then cooled at an ambient temperature in the dark for 15 min. Absorbance was measured at 420 nm using a microplate reader (SpectraMax i3x, Molecular Devices, San Jose, CA, USA). The α-amino acid content was expressed in terms of the calibration curve of the L-leucine (Sigma-Aldrich). The DH was calculated using the following equation:

Degree of hydrolysis (%) = [(L_t_ − L_o_)/ (L_max_ − L_o_)] × 100 (2)

where L_t_ is the amount of leucine equivalence obtained from the sea cucumber hydrolysate, L_o_ is the amount of leucine equivalence in the original sea cucumber, and L_max_ is the maximum amount of leucine equivalence in the rehydrated sea cucumber after hydrolysis using a 6 N HCl solution at 100 °C for 24 h. This likely underestimates the total number of α-amino groups, as all the tryptophan and small amounts of other amino acids might be destroyed, while some dipeptides remain.

#### 3.4.4. DPPH Radical Scavenging Activity

DPPH radical scavenging activity was determined according to the method of Doungapai et al. [8], with a slight modification. An amount of 100 µL of sample solution in distilled water (5 mg/mL) was mixed with 100 µL of 0.05 mmol/L DPPH solution in 95% ethanol. The mixture was incubated at room temperature in the dark for 30 min. The absorbance was obtained at 517 nm using the microplate reader (SpectraMax i3x, Molecular Devices). A DPPH solution without a sample was used as a control, while the 95% ethanol was used as a blank. The DPPH radical scavenging activity of the samples was calculated using the following equation:

DPPH radical scavenging activity (%) = [((A_c_ + A_b_) − A_s_)/A_0_] × 100 (3)

where A_c_, A_b_, and A_s_ are the absorbance of the control, blank, and the sample, respectively.

#### 3.4.5. ABTS Radical Scavenging Activity

ABTS radical scavenging activity was determined according to the method of Upata et al. [13]. The ABTS radical cation was generated by mixing ABTS stock solution (7 mmol/L) with potassium persulfate (2.45 mmol/L) and allowing it to react at room temperature in the dark for 16–18 h. The ABTS solution was diluted with 95% ethanol to an absorbance of 0.70 ± 0.05 at 734 nm. Then, 10 µL of the sample solution (5 mg/mL) was mixed with 190 µL of the ABTS solution, left at room temperature in the dark for 10 min, and then the absorbance was measured at 734 nm using a microplate reader (SpectraMax i3x, Molecular Devices). The ABTS radical scavenging activity of the samples was calculated using the following equation:

ABTS radical scavenging activity (%) = [(A_control_ − A_sample_)/A_control_] × 100 (4)

where A_control_ is the absorbance without the sample and A_sample_ is the absorbance with the sample 

#### 3.4.6. Hydrogen Peroxide (H_2_O_2_) Scavenging Activity

The H_2_O_2_ scavenging activity was determined according to a slightly modified method of Jumeri and Kim [34]. Next, 100 µL of 0.1 mol/L phosphate buffer (pH 5.0) was mixed with 30 µL of the sample solution (5 mg/mL). To the mixture, 20 µL of 10 mmol/L H_2_O_2_ solution was then added and incubated at 37 °C for 5 min. Then, 30 µL of 1.25 mmol/L ABTS and 30 µL of peroxidase (1 unit/mL) were added to the mixture, which was maintained at 37 °C for 10 min. The absorbance was obtained at 405 nm using the microplate reader (SpectraMax i3x, Molecular Devices). The inhibition was expressed using the following equation:

Inhibition (%) = [(H_control_ − H_sample_)/H_control_] × 100 (5)

where H_control_ is the absorbance without the sample and H_sample_ is the absorbance with the sample 

#### 3.4.7. Hydroxyl Radical Scavenging Activity

Hydroxyl radical scavenging activity was measured according to the method of Chi et al. [5], with a slight modification. Then, 1 mL of 1.865 mmol/L 1,10-phenanthroline solution was mixed with 2 mL of the sample solution (5 mg/mL), and 1 mL of 1.865 mmol/L FeSO_4_·7H_2_O solution was added. The reaction was started via the addition of 1.0 mL of 0.03% (*v*/*v*) H_2_O_2_ and incubated at 37 °C for 60 min in a water bath. The absorbance was measured at 536 nm using the microplate reader (SpectraMax i3x, Molecular Devices). The negative control was a mixture without the samples, while the blank mixture did not contain H_2_O_2_. The hydroxyl radical scavenging activity was calculated using the following equation:

Hydroxyl radical scavenging activity (%) = [(A_s_ − A_n_)/(A_b_ − A_n_)] × 100 (6)

where A_s_ is the absorbance of the sample, A_n_ is the absorbance of the negative control, and A_b_ is the absorbance of the blank.

#### 3.4.8. Antiproliferative Activity in the HepG2 Liver Cancer Cell Line 

The antiproliferative activity in the HepG2 liver cancer cell line was determined based on cell viability according to a slightly modified version of the method of Umayaparvathi et al. [35]. For the cell culture, HepG2 cells were seeded in DMEM supplemented with 10% fetal bovine serum (FBS) and 1% penicillin−streptomycin. The cells were maintained at 37 °C and 5% CO_2_ in a humidified atmosphere. The antiproliferative activity of the sea cucumber hydrolysate was evaluated in vitro using a 3-(4,5-dimethylthiazol-2-yl)2,5-diphenyltetrazolium bromide (MTT) assay. The cytotoxicity of the hydrolysate on the HepG2 liver cancer cell line was determined and expressed as cell viability. The HepG2 cells were seeded in 96-well plates at a cell density of 1 × 10^4^ cells/well at 37 °C and 5% CO_2_ in a humidified atmosphere. After 24 h of incubation, the cells were treated and untreated (control) with 100 µL of the sea cucumber hydrolysate (5 mg/mL) and incubated overnight (24 h) at 37 °C in a humidified 5% CO_2_ atmosphere. Subsequently, 100 µL of fresh DMEM and 20 µL of MTT (5 mg/mL in phosphate-buffered saline) were added into each well and incubated for 4 h. The DMEM was then aspirated and replaced with 100 µL of DMSO to solubilize the colored products for 10 min. The optical density (OD) was measured using a microplate reader (Synergy H4 Hybrid, BioTek, Shoreline, WA, USA) at 540 and 630 nm. The cell viability (%) of HepG2 cells was calculated using the following equation:

Cell viability (%) = [OD_treated_/OD_control_] × 100 (7)

where OD_treated_ = (OD of treated at 540 nm—OD of treated at 630), and OD_control_ = (OD of control at 540 nm—OD of control at 630 nm). All tests were performed in triplicate and the average values were recorded.

### 3.5. The Effect of the Sea Cucumber Hydrolysate Concentration on the Antiproliferative Activity of the HepG2 Liver Cancer Cell Line

The effect of the sea cucumber hydrolysate concentration on the antiproliferative activity in the HepG2 liver cancer cell line was evaluated using the MTT assay. The various concentrations (0.05–5.0 mg/mL) of the sea cucumber hydrolysate were treated in HepG2 cells. The antiproliferative activity in the HepG2 liver cancer cell line was determined according to the above-mentioned method (Section 3.4.7) and shown as the HepG2 cell viability (%).

### 3.6. Experimental Design and Statistical Analysis

The optimization of sea cucumber hydrolysis conditions was determined using response surface methodology with a two-factor, three-level, face-centered central composite design. The two factors of hydrolysis time (X_1_) and enzyme concentration (X_2_) were optimized with coded values at three levels, of −1, 0, and +1. The responses of the experiment were DH (%), yield (%), DPPH radical scavenging activity (%), ABTS radical scavenging activity (%), hydrogen peroxide scavenging activity (%), hydroxyl radical scavenging activity (%), and antiproliferative activity on the HepG2 liver cancer cell line (%). The 11 treatments were generated to include 8 incomplete factorial points and 3 replicated central points. The experimental design, data analysis, and response surface plots were created using Design Expert software version 11 (Stat-Ease, Inc., Minneapolis, MN, USA). Complete quadratic mathematical models with real variables were established for each response using the following equation:

Yi = A_0_ + ΣA_i_X_i_ + ΣA_j_X_j_ + ΣA_ii_X_i_^2^ + Σ A_jj_X_j_^2^ + ΣΣ A_ij_X_i_X_j_ (8)

where Y_i_ represents the predicted response. X_i_ and X_j_ are the codes for the factors or independent variables, whereas A_0_, A_i_, A_j_, A_ii_, A_jj_, and A_ij_ are the constants and coefficients for the linear, quadratic, and interaction parameters, respectively. 

One-way analysis of variance was used to determine significant differences (*p* ≤ 0.05) using the Statistical Package for the Social Sciences (SPSS) software, version 17.0 (SPSS Inc., Chicago, IL, USA). Duncan’s new multiple-range tests were used to test for the differences between means (*p* ≤ 0.05). All experiments and data are presented as the mean ± standard deviation of three replications.

## 4. Conclusions

The present study investigated the enzymatic protein hydrolysis of the sea cucumber body wall for the production of a protein hydrolysate containing highly bioactive properties, which could be applied as an effective antiproliferative effect on cancer cells. The optimal conditions were determined for the preparation of the sea cucumber hydrolysate using papain hydrolysis to generate a high yield and effective antioxidant and antiproliferative effects on cancer cells’ properties. Complete quadratic models were fitted for all dependent variables. The optimum conditions for preparing the sea cucumber hydrolysate from multiple responses were a 4.3% papain concentration and a hydrolysis time of 360 min. The obtained sea cucumber hydrolysate could have potential to be developed as a functional ingredient for natural antioxidant and anticancer properties. Therefore, additional research on the peptide purification, identification, and anticancer mechanisms of the sea cucumber hydrolysate should be further studied.

## Figures and Tables

**Table 1:** Chemical composition of dried and rehydrated sea cucumber.

Samples	Moisture (%)	Protein (%)	Fat (%)	Ash (%)
Dried sea cucumber	17.22 ± 0.47	65.75 ± 0.78	1.41 ± 0.08	13.98 ± 0.03
Sea cucumber Slurry	82.69 ± 0.10	12.40 ± 1.3	1.24 ± 0.10	2.03 ± 0.09

**Table 2:** The eleven experimental units and seven responses for the preparation of the sea cucumber hydrolysate using papain hydrolysis.

Experimental Units	Factors		Responses						
	X_1_	X_2_	Yield (%)	DH (%)	DPPH (%)	ABTS (%)	H_2_O_2_ (%)	OH· (%)	Cell Viability (%)
	(min)	(%)							
1	60	1	6.69 ^a^ ± 1.06	79.6 ^a^ ± 0.44	89.5 ^bcd^ ± 0.84	66.6 ^bcde^ ± 3.14	21.3 ^ab^ ± 1.53	78.1 ^bc^ ± 3.77	5.14 ^d^ ± 0.55
2	360	1	10.9 ^bc^ ± 1.98	81.1 ^ab^ ± 1.74	82.5 ^abc^ ± 1.12	59.9 ^a^ ± 0.74	38.8 ^d^ ± 0.71	82.5 ^c^ ± 1.66	3.06 ^b^ ± 0.42
3	60	5	11.7 ^cd^ ± 0.23	79.8 ^a^ ± 0.34	79.8 ^a^ ± 1.97	63.9 ^abc^ ± 1.20	27.0 ^c^ ± 2.68	82.5 ^c^ ± 2.33	4.93 ^d^ ± 0.46
4	360	5	12.8 ^e^ ± 0.04	85.2 ^bc^ ± 0.95	91.7 ^cd^ ± 1.68	69.2 ^e^ ± 2.54	41.7 ^d^ ± 2.96	84.6 ^bc^ ± 4.01	3.77 ^bc^ ± 0.49
5	60	3	9.96 ^b^ ± 0.31	80.6 ^bc^ ± 1.99	85.3 ^ab^ ± 1.12	66.1 ^abcd^ ± 1.94	25.6 ^c^ ± 0.73	80.1 ^bc^ ± 1.39	4.81 ^cd^ ± 0.45
6	360	3	12.7 ^e^ ± 0.03	84.7 ^c^ ± 2.17	88.1 ^bcd^ ± 3.25	64.3 ^cde^ ± 0.85	38.9 ^d^ ± 0.85	83.0 ^bc^ ± 1.90	2.61 ^a^ ± 0.42
7	210	1	9.59 ^b^ ± 0.35	83.1 ^bc^ ± 0.97	87.2 ^bcd^ ± 3.08	61.6 ^ab^ ± 2.55	18.7 ^a^ ± 14.84	76.2 ^b^ ± 3.15	3.98 ^cd^ ± 0.62
8	210	5	12.4 ^de^ ± 0.23	84.5 ^bc^ ± 2.16	88.4 ^bcd^ ± 1.68	69.3 ^de^ ± 0.92	28.8 ^c^ ± 1.06	83.4 ^bc^ ± 0.92	4.01 ^bc^ ± 0.85
9	210	3	12.3 ^de^ ± 0.13	83.3 ^bc^ ± 3.81	91.7 ^d^ ± 0.56	67.6 ^de^ ± 0.10	25.8 ^c^ ± 1.40	73.0 ^a^ ± 7.59	4.77 ^cd^ ± 0.16
10	210	3	11.1 ^bc^ ± 0.78	85.1 ^bc^ ± 0.93	87.5 ^bcd^ ± 1.40	66.7 ^bcde^ ± 1.57	25.6 ^bc^ ± 1.92	76.7 ^bc^ ± 4.55	3.98 ^bc^ ± 0.53
11	210	3	12.3 ^de^ ± 2.29	82.8 ^ab^ ± 1.80	88.9 ^bcd^ ± 0.69	66.7 ^bcde^ ± 2.12	27.0 ^c^ ± 1.92	76.0 ^b^ ± 3.98	4.20 ^bc^ ± 0.24

**Note:** X_1_ is the hydrolysis time (min) and X_2_ is the concentration of papain (%), DPPH is the DPPH radical scavenging activity (%), ABTS is the ABTS radical scavenging activity (%), H_2_O_2_ is the hydrogen peroxide scavenging activity (%), and OH· is the hydroxyl radical scavenging activity (%). Different letters in the same column indicate statistical differences (*p* ≤ 0.05).

**Table 3:** Response surface model for the papain-hydrolyzed sea cucumber hydrolysate.

Responses	Quadratic Polynomial Model	*R* ^2^	*p*-Value for Models	*p*-Value for Lack of Fit
Yield (%)	Y_1_ = 11.9 + 1.35X_1_ + 1.59X_2_ − −0.79X_1_X_2_ − 0.52X_1_^2^ − 0.86X_2_^2^	0.9643	0.0013	0.9654
DH (%)	Y_2_ = 84.0 + 1.84X_1_ + 0.96X_2_ + 0.97X_1_X_2_ − 1.76X_1_^2^ − 0.61X_2_^2^	0.9023	0.0146	0.8619
DPPH (%)	Y_3_ = 89.2 + 1.29X_1_ − 0.10X_2_ + 4.72X_1_X_2_ − 2.26X_1_^2^ − 1.20X_2_^2^	0.9225	0.0083	0.9633
ABTS (%)	Y_4_ = 66.8 − 0.54X_1_ + 2.38X_2_ + 2.99X_1_X_2_ − 1.17X_1_^2^ − 0.88X_2_^2^	0.9023	0.0145	0.1068
H_2_O_2_ (%)	Y_5_ = 25.6 − 7.58X_1_ + 3.13X_2_ − 0.70X_1_X_2_ + 7.33X_1_^2^ − 1.14X_2_^2^	0.9661	0.0011	0.0925
OH· (%)	Y_6_ = 76.2 + 1.56X_1_X + 0.28X_2_ − 0.59X_1_X_2_ + 4.10X_1_^2^ + 2.34X_2_^2^	0.8354	0.0495	0.5691
Cell viability (%)	Y_7_ = 4.14 − 0.91X_1_ − 0.09X_2_ + 0.23X_1_X_2_ − 0.17X_1_^2^ + 0.12X_2_^2^	0.8525	0.0384	0.4981

**Note:** X_1_ is the hydrolysis time (min) and X_2_ is the concentration of papain (%), DPPH is the DPPH radical scavenging activity (%), ABTS is the ABTS radical scavenging activity (%), H_2_O_2_ is the hydrogen peroxide scavenging activity (%), and OH· is the hydroxyl radical scavenging activity (%).

**Table 4:** The constants, coefficients, and *p*-values of independent variables and interactions for all responses of sea cucumber hydrolysis with papain.

Responses	Code	Intercept	X_1_	*p*-Value	X_2_	*p*-Value	X_1_X_2_	*p*-Value
Yield (%)	Y_1_	11.872	1.346	0.001	1.594	0.001	−0.789	0.023
DH (%)	Y_2_	83.997	1.841	0.005	0.957	0.055	0.971	0.093
DPPH scavenging activity (%)	Y_3_	89.188	1.291	0.077	0.099	0.871	4.715	0.001
ABTS scavenging activity (%)	Y_4_	66.728	−0.539	0.359	2.384	0.007	2.994	0.006
H_2_O_2_ scavenging activity (%)	Y_5_	25.636	7.584	0.0001	3.125	0.011	−0.697	0.507
OH· scavenging activity (%)	Y_6_	76.137	1.557	0.043	2.278	0.050	−0.593	0.612
Cell viability (%)	Y_7_	4.142	−0.906	0.004	0.091	0.625	0.228	0.335

**Note:** X_1_ is the hydrolysis time (min) and X_2_ is the concentration of papain (%).

**Table 5:** Parameter setting for multi-response optimization, composite desirability, and predicted and experimental values of all responses.

Response	Parameters					Predicated Value	Real Value	Composite Desirability
	Goal	Lower	Upper	Weight	Importance			
X_1_	is in range	60	360	1	3	360	360	0.92
X_2_	is in range	1	5	1	3	4.3	4.3	
Yield (%)	maximize	6.69	12.75	1	3	12.87	12.10 ± 0.54	
DH (%)	maximize	79.58	85.22	1	3	85.07	74.52 ± 1.06	
DPPH (%)	maximize	79.75	91.66	1	3	90.83	89.74 ± 1.51	
ABTS (%)	maximize	59.88	69.33	1	3	68.12	74.92 ± 0.81	
H_2_O_2_ (%)	maximize	18.66	41.7	1	3	41.64	39.42 ± 1.74	
OH· (%)	maximize	73.03	84.56	1	3	84.3	88.71 ± 1.49	
Cell viability (%)	minimize	2.61	5.14	1	3	3.32	9.89 ± 1.37	

**Note:** X_1_ is the hydrolysis time (min) and X_2_ is the concentration of papain (%), DPPH is the DPPH scavenging activity (%), ABTS is the ABTS scavenging activity (%), H_2_O_2_ is the H_2_O_2_ scavenging activity (%), and OH· is the hydroxyl radical scavenging activity (%).

## Data Availability

Data are reported in the article.
